# Emergency Department Presentations for Injuries Following Agency-Notified Child Maltreatment: Results From the Childhood Adversity and Lifetime Morbidity (CALM) Study

**DOI:** 10.1177/10775595241264009

**Published:** 2024-06-20

**Authors:** Mike Trott, Claudia Bull, Urska Arnautovska, Dan Siskind, Nicola Warren, Jake M. Najman, Steve Kisely

**Affiliations:** 1 Princess Alexandra Hospital Southside Clinical Unit, Greater Brisbane Clinical School, Medical School, 1974 The University of Queensland, Brisbane, QLD, Australia; 2 Metro South Addiction and Mental Health Service, Brisbane, QLD, Australia; 3 ALIVE National Centre for Mental Health Research Translation, Brisbane, QLD, Australia; 4 School of Public Health, 420004 The University of Queensland, Brisbane, QLD, Australia; 5 School of Social Sciences, 1974 The University of Queensland, Brisbane, QLD, Australia; 6 Departments of Psychiatry, Community Health and Epidemiology, Dalhousie University, Halifax, NS, Canada

**Keywords:** child abuse, cohort studies, domestic/intimate partner violence, epidemiology, injury prevention

## Abstract

Child maltreatment (CM) is associated with negative health outcomes in adulthood, including deliberate self-harm (DSH), suicidal behaviours, and victimisation. It is unknown if associations extend to emergency department (ED) presentations for non-DSH related injuries. Birth cohort study data was linked to administrative health data, including ED presentations for non DSH related injuries and agency-reported and substantiated notifications for CM. Adjusted analyses (*n* = 6087) showed that any type of agency-reported notification for CM was significantly associated with increased odds of ED presentation for injuries (aOR = 1.57; 95% CI 1.32–1.87). In moderation analyses, women yielded significantly higher odds of notified and substantiated physical abuse, substantiated emotional abuse, and being subject to more than one type of substantiated abuse than males. ED presentations for injuries could be a proxy for risky behaviours, disguised DSH/suicidal behaviours, or physical abuse. The consistent findings in women may point to victimisation via interpersonal violence.

## Introduction

Injury-related presentations to the Emergency Department (ED) are common, with unintentional falls being the most frequent cause of injury in the United States (US) in all age groups except for ages 10–24 years old (Weiss et al., 2018). Elsewhere, the Australian Institute of Health and Welfare (AIHW) reported that of the 8.8 million ED presentations made in 2022–2023, roughly 2 million (22%) were for injury, poisoning and other consequences of external causes (ICD-10-AM codes S00-T98) (Australian Institute of Health and Welfare, 2023). Although the exact cause of injuries is not routinely reported, they can be broadly categorised into three groups: (a) suicidal behaviours with the intent to die; (b) deliberate self-harm (DSH), encompassing direct injury to ones-self without conscious suicidal intent; and (c) non-DSH related injuries, encompassing instances of harm or injury that occur without clear intent on the part of the presenting individual (such as accidents or assaults by another person) and without the clear purpose to cause self-harm or suicide.

Child maltreatment (CM) can be defined as all types of physical and/or emotional mistreatment, sexual abuse, neglect, or negligent care, as well as commercial forms of exploitation, leading to actual or potential harm to a child’s health, well-being, or self-worth (World Health Organization, 2006). It is frequently stratified into four sub-types: physical abuse, emotional abuse, sexual abuse, and neglect (World Health Organization, 2020). Between 30 and 75% of children experience some kind of CM (Mathews et al., 2023; World Health Organization, 2020), and being subject to more than one type of maltreatment is more common than experiencing a single type (Higgins et al., 2023). Girls are also more likely to experience CM than boys (Felitti et al., 1998; Mathews et al., 2023; World Health Organization, 2020). CM is associated with negative health outcomes in adulthood. Several studies have reported that people who reported CM in their youth were more likely to be diagnosed with mental health conditions in later life (Scott et al., 2023; Strathearn et al., 2020a). CM has also been consistently associated with ED presentations for suicidal behaviours and DSH (Fliege et al., 2009; Gratz, 2003). These outcomes, however, have been reported to differ according to sex. For example, antisocial behaviour is significantly associated with CM in males, but not females, while the reverse has been reported for intravenous drug use (Strathearn et al., 2020a). Similarly, only females have been shown to yield significant associations between self-reported CM and psychosis in sex-stratified studies (Fisher et al., 2009). Moreover, Kisely et al., 2024a reported that associations between CM and being victim to intimate partner violence in later life were stronger in females than in males. Evidently, to best support tailored treatment plans and healthcare intervention, there is a clear need to understand why men and women differ in terms of their CM life course outcomes.

One outcome that has been explored less in the literature is the relationship between CM and injuries not relating to DSH/suicidal behaviours, subsequently referred to as ‘injuries’. One of the few studies in this area reported that agency-reported CM notifications were associated with an increased risk of ED presentations for ‘injury and accidents’ (which excluded presentations for self-harm) from childhood up to the age of 21 (Gnanamanickam et al., 2022). There are several hypotheses that could explain a possible link between CM and injuries. For example, there are significant associations between CM and being the victim of physical assaults and/or domestic violence in later life (Noll, 2005; Ogloff et al., 2012), as well as engaging in more risky and violent behaviours (Briere & Elliott, 2003), all of which may result in ED presentations for injuries. Furthermore, it is possible that some ED presentations for injury are undisclosed, or differently coded, DSH. For example, a self-harming behaviour such as wrist cutting may be coded as a variety of S51 sub-codes (open wound of elbow and wrists) (Fliege et al., 2006; Fliege et al., 2002; Kocalevent et al., 2005).

Child maltreatment studies frequently rely on self-reported measures to identify people who have experienced CM. However, retrospective self-reports of CM may not encompass maltreatment as captured by other sources such as contemporaneous notifications to statutory agencies (Newbury et al., 2018). One method of obtaining this non-subjective data regarding CM and its consequences over the life course, is to link data from child protection systems to administrative health datasets. This can reveal patterns in health outcomes, service use, and sociodemographic information across populations and over time. The previously cited study of the association between agency-reported CM and ED presentations for injury from childhood into early adulthood is one example (Gnanamanickam et al., 2022). We therefore applied a similar methodology to investigate associations between CM and ED presentations for injuries in a secondary dataset in adults from 25 to 39 years. We also examined if there were differences by CM sub-categories and gender. We aimed to establish if there was an association between CM, including sub-types, and ED presentations for injuries.

In line with the results from previous studies in early adulthood, we hypothesised that CM would be positively associated with injuries, and that all sub-categories of CM would be associated with subsequent ED presentations for injuries in both men and women.

## Methods

Ethical approval was granted by The University of Queensland Human Research Ethics Committee (HREC) (2021/HE001925) and the Metro South Health HREC (HREC/2022/QMS/83690). We also prospectively registered a pre-trial protocol with the Australian and New Zealand Clinical Trials Registry (ACTRN12622000870752). Reporting of this study followed STrengthening the Reporting of OBservational studies in Epidemiology (STROBE) guidance (Von Elm et al., 2007), and the REporting of studies Conducted using Observational Routinely-collected health Data (RECORD) Statement (Benchimol et al., 2015).

### Participants

This study used secondary data from the Childhood Adversity and Lifetime Morbidity (CALM) study dataset (Kisely et al., 2023). The CALM study linked the Mater-University of Queensland Study of Pregnancy (MUSP) birth cohort with 40 years of follow up to Queensland-wide administrative health datasets, including ED presentations (Kisely et al., 2023; Najman et al., 2015). The MUSP study recruited *n* = 6753 women and their resultant *n* = 7223 children born at the Mater Mothers Hospital, the principal obstetrics unit for Brisbane, between 1981 and 1983 (Najman et al., 2015).

### Data Sources and Linkage

In September 2000, notifications/substantiations of CM to the Department of Families, Youth and Community Care were anonymously linked to MUSP cohort records (Strathearn et al., 2020b). Reports were substantiated if, after investigation by child protective services, there was a reasonable belief of a case of CM. In 2023, administrative health data from Queensland Health were anonymously liked to the MUSP database to create the MUSP-CALM dataset.

**Figure 1.**
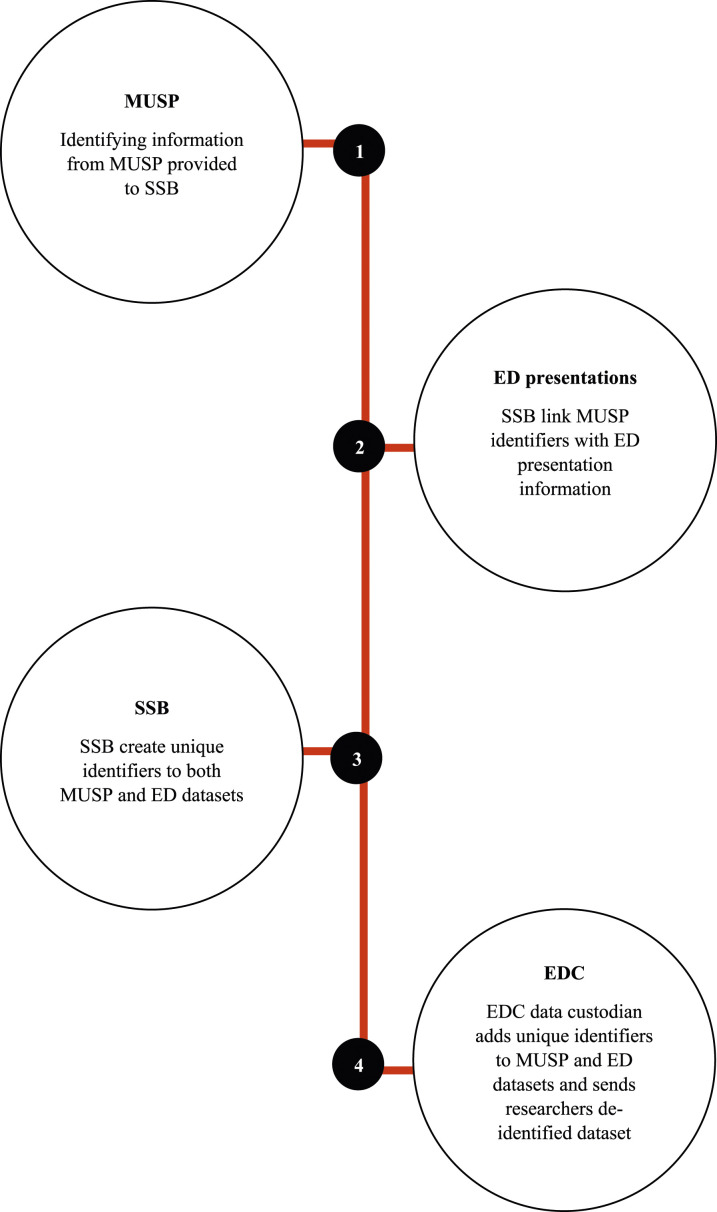
Flowchart showing administrative data linkage process.

The data linkage was conducted by the Statistical Services Branch (SSB) of Queensland Health, with the MUSP data custodian providing a dataset containing only names, birth dates, and sex of the birth cohort participants to the SSB. Within the SSB, Oracle software was used to match these details with the Queensland Health Master Linkage File. The SSB subsequently assigned a unique key to each linked record and shared this with the Emergency Data Collection data custodian, who used it as an identifier for the linked records that were sent to the researchers (with no personal identifiers). This yielded a dataset that included MUSP variables, and information on all Queensland public ED presentations from when the participants were 25–39 years of age. The ED presentations information included the ICD-10-AM codes, identifying reasons for any respective presentation. Information on the accuracy and quality of data linkage is available via the Queensland Data Linkage Framework (State of Queensland, 2021). The process of data linkage is graphically represented in Figure 1.

### Statistical Analysis and Data Preparation

For the purposes of this study, injuries included the following ICD-10-AM codes: injuries to the head (S00-S09); neck (S10-S19); thorax (S20-S29); abdomen (S30-S39); shoulders and upper arms (S40-S49); elbows, forearms, and hands (S50-S69); hips, legs, and feet (S70-S99); injuries involving multiple body regions (T07); foreign bodies (T15-T19), injuries of unspecified body regions (T14), burns (T20-T32), poisoning (T36-T50). ICD-10-AM codes that have been reported as being associated/coded in the ED as potential DSH or suicidal behaviours were excluded (Kisely et al., 2024b; Sveticic et al., 2020). These included codes related to DSH (S51.9, T00.9), suicidal ideation (T50.9), and suicide attempt (T40.0, T44.3, T71, T44.7, T43.69, T45, T39.3, T39.1, T42.4, T43.9, and T43.5).

All analyses were performed using SPSS (Version 29). As data for ED presentations was not normally distributed, dichotomous variables indicating ‘ever’ versus ‘never’ having presented for injuries, were created. To determine if previous CM was associated with ED presentations for injuries, bivariate logistic regressions were conducted. Missing data ranged from 0.03% for all substantiated CM sub-types, to 0.08% for all notified CM sub-types, which was considered negligible, therefore missing cases were deleted listwise in all analyses. All analyses were sub-grouped according to the type of CM notification (agency-reported or substantiated), and by sub-types of CM (physical, emotional, sexual abuse, and neglect). We also created a category called “more than two types” if individuals ever had notifications/substantiations for two or more types of CM (e.g. physical *and* emotional abuse) up to the age of 15. Multivariate analyses were then conducted, adjusting for baseline sex, family income, parental relationship status, and ethnicity, as these have been shown to be associated with CM and/or psychiatric symptoms in previous research from the same dataset (Kisely et al., 2020). To determine differences between baseline sex, interaction terms between baseline sex and the respective outcome variable were created and imputed as covariates into multivariate models (e.g. for the notified physical abuse sub-group, the interaction term was notified physical abuse*baseline sex). Bivariate and multivariate sub-group analyses according to baseline sex were then conducted, with multivariate analyses being controlled for family income, parental relationship status, and ethnicity.

To determine the robustness of results, a pre-planned propensity score analysis was conducted as a sensitivity analysis for missing data. For this, a covariate that respectively represented baseline characteristics (sex, family income, parental relationship and ethnicity for non-sex-stratified analyses, and family income at time of birth, parental relationship, and ethnicity for the sex-stratified analysis) was created using logistic regression for the entire baseline MUSP cohort (*n* = 7214). This meant that the sensitivity analysis considered the demographic information of all original MUSP cohort participants; not just those whose administrative health data could be linked (*n* = 6087). This approach was preferred to undertaking multiple imputation because it could not be assumed that data were missing at random.

## Results

Of the 7214 participants in the baseline MUSP dataset, 6087 participants (84.4%) were successfully linked to the administrative health data. Full demographic details can be found in Table 1. In brief, 2944 (48.4%) were female, 323 (5.3%) identified as Aboriginal and/or Torres Strait Islanders, and 1840 (30.2%) had a family income of $10,399 or less (the financial threshold for poverty in 1981). A total of 2246 participants (36.9%) presented to the ED with injuries at least once between the ages of 25–39 years. Regarding CM, 609 people (10.0%) had any kind of CM notification, of which 389 (6.4% of total sample; 63.9% of notified CM) were substantiated. Regarding agency-reported sub-types, neglect was the most common, followed by physical abuse, emotional abuse, with sexual abuse notifications being the least prevalent. The most common sub-type of substantiated CM was physical, followed by emotional, neglect, and sexual. Women had significantly higher rates of any agency-reported CM, as well as agency-reported and substantiated sexual abuse. All other types of CM were not different between sex, see Table 1.

**Table 1.** Demographic Variables of the Overall Sample and Stratified by Baseline Sex.

	Overall		Male		Female		*p* Between Sex^†^
	*n*	%	*n*	%	*n*	%	
	6087	100.0	3143	51.6	2944	48.4	-
Identified as indigenous	323	5.3	172	5.5	151	5.1	0.55
Parents not living together at birth	778	12.8	394	12.5	384	13.0	0.55
Family income prior to birth ≤$10,399/year	1840	30.2	894	28.4	946	32.1	**0.002**
Presentations for injuries	2246	36.9	1360	60.6	886	39.4	**<0.001**
Presence of any CM notifications	609	10.0	289	9.2	320	10.9	**0.03**
Notified physical abuse	359	5.9	185	5.9	174	5.9	0.97
Notified emotional abuse	333	5.5	166	5.3	167	5.7	0.50
Notified neglect	364	6.0	184	5.9	180	6.1	0.67
Notified sexual abuse	197	3.2	54	1.7	143	4.9	**<0.001**
Notifications for more than two sub-types of abuse	395	6.5	196	6.2	199	6.8	0.41
Presence of any CM substantiated notifications	389	6.4	189	6.0	200	6.8	0.21
Substantiated physical abuse	204	3.4	107	3.4	97	3.3	0.81
Substantiated emotional abuse	199	3.3	97	3.1	102	3.5	0.41
Substantiated neglect	192	3.2	97	3.1	95	3.2	0.76
Substantiated sexual abuse	109	1.8	27	1.0	82	2.8	**<0.001**
More than two sub-types of substantiated abuse	210	3.4	97	3.1	113	3.8	0.11

^†^*p*-values from Chi-square tests; CM = child maltreatment. Bold indicates statistically significant results.

The pooled analyses yielded several significant associations between ED presentations and CM. Specifically, the adjusted odds ratio (aOR) for any agency reported CM was 1.57 (95% CI 1.32–1.87), notified physical abuse 1.56 (95% CI 1.25–1.94), notified emotional abuse 1.54 (95% CI 1.23–1.93), notified sexual abuse 1.43 (95% CI 1.07–1.92), notified neglect 1.62 (95% CI 1.30–2.01), and notified for two or more types of CM 1.58 (95% CI 1.28–1.95). aORs for substantiated CM in the overall sample were similar, with any agency substantiated CM = 1.41 (95% CI 1.14–1.75), substantiated physical abuse 1.47 (95% CI 1.10–1.95), substantiated emotional abuse 1.52 (95% CI 1.14–2.03), substantiated neglect 1.60 (95%CI 1.19–2.15), substantiated sexual abuse 1, and substantiated for two or more types of CM 1.41 (95% CI 1.06–1.87). Full results can be found in Table 2.

**Table 2.** Odds of ED Presentations for Injury Following Previous CM.

	Overall (*n* = 6087)			Male (*n* = 3143)			Female (*n* = 2944)		
	OR (95%CI)	aOR (95%CI)^a^	*p*	OR (95%CI)	aOR (95%CI)^b^	*p*	OR (95%CI)	aOR (95%CI)^b^	*p*
Any agency-reported notifications of CM	1.58 (1.33–1.87)	1.57 (1.32–1.87)	**<0.001**	1.42 (1.12–1.81)	1.40 (1.09–1.78)	**0.008**	1.87 (1.48–2.38)	1.74 (1.37–2.22)	**<0.001**
Physical abuse^c^	1.62 (1.31–2.01)	1.56 (1.25–1.94)	**<0.001**	1.26 (0.94–1.70)	1.23 (0.91–1.66)	0.174	2.18 (1.60–2.97)	1.99 (1.45–2.73)	**<0.001**
Emotional abuse	1.59 (1.27–1.98)	1.54 (1.23–1.93)	**<0.001**	1.37 (1.00–1.87)	1.34 (0.98–1.83)	0.071	1.93 (1.40–2.64)	1.77 (1.29–2.45)	**<0.001**
Neglect	1.67 (1.35–2.07)	1.62 (1.30–2.01)	**<0.001**	1.57 (1.16–2.12)	1.54 (1.14–2.08)	**0.005**	1.85 (1.36–2.52)	1.69 (1.24–2.31)	**<0.001**
Sexual abuse	1.28 (0.96–1.71)	1.43 (1.07–1.92)	**0.017**	1.32 (0.77–2.26)	1.29 (0.75–2.22)	0.354	1.58 (1.12–2.23)	1.48 (1.04–2.09)	**0.029**
Two or more sub-types	1.62 (1.32–1.99)	1.58 (1.28–1.95)	**<0.001**	1.40 (1.05–1.87)	1.37 (1.02–1.83)	**0.036**	1.98 (1.48–2.65)	1.82 (1.35–2.45)	**<0.001**
Any substantiated notifications of CM	1.45 (1.18–1.78)	1.41 (1.14–1.75)	**0.001**	1.26 (0.94–1.69)	1.23 (0.92–1.66)	0.168	1.75 (1.31–2.35)	1.60 (1.19–2.16)	**0.002**
Physical abuse^c^	1.54 (1.17–2.04)	1.47 (1.10–1.95)	**0.009**	1.16 (0.79–1.70)	1.13 (0.76–1.66)	0.548	2.16 (1.44–3.24)	1.96 (1.30–2.96)	**0.001**
Emotional abuse^c^	1.56 (1.17–2.07)	1.52 (1.14–2.03)	**0.005**	1.19 (0.79–1.79)	1.16 (0.77–1.74)	0.484	2.13 (1.43–3.16)	1.98 (1.32–2.95)	**<0.001**
Neglect	1.67 (1.25–2.23)	1.60 (1.19–2.15)	**0.002**	1.35 (0.90–2.03)	1.32 (0.88–1.99)	0.176	2.15 (1.43–3.24)	1.91 (1.25–2.90)	**0.003**
Sexual abuse	1.03 (0.70–1.53)	1.15 (0.77–1.71)	0.487	0.79 (0.42–1.95)	0.89 (0.41–1.92)	0.766	1.35 (0.86–2.13)	1.23 (0.78–1.96)	0.375
Two or more sub-types^c^	1.43 (1.08–1.89)	1.41 (1.06–1.87)	**0.018**	1.09 (0.73–1.64)	1.06 (0.71–1.60)	0.775	1.97 (1.35–2.87)	1.79 (1.22–2.63)	**0.003**

ED = Emergency Department; OR = Odds Ratio; aOR = adjusted odds ratio; CM = Childhood Maltreatment.

^a^Adjusted for gender, ethnicity, family income at birth, and family living arrangement at birth.

^b^Adjusted for ethnicity, family income at birth, and family living arrangement at birth.

^c^Indicates significant moderation of baseline sex.

Moderation analyses found that females had significantly higher odds of notified physical abuse than men (aOR 1.99; 95% CI 1.45–2.73 vs. 1.23; 95% CI 0.91–1.66, respectively, *p* = .004). Females also had higher odds of substantiated physical abuse (aOR 1.96; 95% CI 1.30–2.96 for females vs. 1.13; 95% CI 0.76–1.66 for males, *p* = .024), substantiated emotional abuse (aOR 1.98; 95% CI 1.32–2.95 for females vs. 1.16; 95% CI 0.77–1.74 for males, *p* = .023), and having substantiated CM across two or more CM sub-types (aOR 1.79; 95% CI 1.22–2.63 for females vs. 1.06; 95% CI 0.71–1.60 for males, *p* = .033). For full results, see Table 2.

Sensitivity analyses revealed no changes in the magnitude or significance of results, see Supplementary Table 1.

## Discussion

This study examined associations between CM and subsequent ED presentations for injury not related to deliberate self-harm, stratified and moderated by sex. Significant associations were found across several types of CM, including a 65% increase in the odds of ever presenting to the ED from 25-39 years of age with injuries if a person had any type of previous agency-reported notifications of CM. If these notifications were substantiated, the odds remained 51% higher. These associations were similar (in effect size and significance) when stratified by CM sub-type, with the exception substantiated sexual abuse, which was not associated with ED presentations. When stratified by sex at birth, only women yielded consistently significant odds across any CM and all sub-types, despite there being no significant bivariate sex differences in the number of cases of both agency-reported and substantiated CM. Furthermore, women were significantly more likely to present with injuries than men if they had notified or substantiated physical abuse, substantiated emotional abuse, or substantiation of two or more sub-types of CM.

### Overall Results

Although this is the first study to our knowledge that examined associations between childhood CM and ED presentations between the ages of 25–39 for injuries, the results concur with other studies of agency-reported CM and ED presentations for injury in younger cohorts. Gnanamanickam et al. (2022), who used administratively linked data in South Australia to examine ED presentations in adults 18–25, reported that the incident rate ratio (IRR) for ED presentations related to ‘injury and accidents’ (excluding self-harm and including ICD-10 codes S-T) in adults aged 18–25 were twice as high for people who had agency-reported CM; a similar result to our findings. Gnanamanickam et al. also reported a higher IRR for substantiated cases of CM, whereas the current study found a similar effect sizes across agency-reported and substantiated CM in the overall analysis. It is possible that the differences between the effect sizes found in the earlier study and ours is because of the age groups examined (18–25 years for Gnanamanickam et al. and 25–39 for our study). For example, the AIHW reported that types of ED presentations for injuries and poisoning change throughout the life course, representing 30% of all 2022–2023 ED presentations in ages 15–24 versus 22% and 21% in ages groups 25–34 and 35–44, respectively (Australian Institute of Health and Welfare, 2023). Research is warranted to explore this further. Our overall, non-sex stratified, results also concur with Hussey et al. (Hussey et al., 2005), who argued that behavioural and developmental outcomes for children with agency-reported versus substantiated CM are not significantly different.

Although it has been well-reported that experiencing CM is associated with DSH in later life (Fliege et al., 2009; Kisely et al., 2024b), this study has added to the evidence base in that this association also extends to injuries not clearly related to DSH. More broadly, it adds evidence that CM has wide-ranging implications for later life, except in the case of substantiated sexual abuse. The finding that substantiated sexual abuse was not associated with injuries was surprising, as previous sexual abuse has been consistently associated with several negative outcomes in adulthood, including substance misuse, suicide attempts, sexual revictimization, and non-suicidal self-injury (Hailes et al., 2019). It is possible that the small sample size for substantiated sexual abuse (*n* = 109) meant our analysis lacked the statistical power to yield significant results.

### Sex-Stratified Results

Surprisingly, we found more consistent associations for women but not for men across analyses, and we found that women are significantly more likely to present to the ED after experiencing notified or substantiated physical abuse, substantiated emotional abuse, or substantiation for two or more types of CM. Although differences by sex have been noted for other CM related outcomes, results have been largely mixed. For instance, antisocial behaviour at 21 years of age was significantly associated with CM in males, but not females, while the reverse was true for intravenous drug use (Strathearn et al., 2020a). Conversely, only women were found to yield significant associations between self-reported CM and psychosis (Fisher et al., 2009). This study adds to the literature that outcomes related to previous CM differ by sex and adds more evidence that future studies should (a) stratify by sex, and (b) examine possible reasons for these differences. The strong associations between ED presentations for injuries and CM seen in the current study, particularly in women, may be multifaceted. Previous research illustrates the impact of intragenerational transmission of violence. That is, women who experienced CM are subsequently more likely to experience domestic violence and victimisation in adulthood which may contribute to the higher odds of presenting to ED with injuries seen in the current study (Anderson et al., 2016; Coid et al., 2001; Noll, 2005; Ogloff et al., 2012; Renner & Slack, 2006). Another possibility is that CM may increase the likelihood that an individual engages in risky, impulsive behaviours that could lead to ED presentations independent of DSH (Madge et al., 2011). Moreover, our results may reflect sex differences in health-seeking behaviour: women have been consistently reported to have be more likely to seek medical help than men, including in studies using similar cohorts (Deeks et al., 2009; Thompson et al., 2016). Further exploration of these hypotheses are warranted.

### Strengths and Implications

One strength of this study is that participants were at least 25 years old upon ED presentation, thereby eliminating the possibility that visits preceded instances of CM. Moreover, by stratifying according to CM sub-types and sex, this study offers unique and comprehensive insights into the detrimental effects of CM and has several implications for policy and practitioners. First, our findings highlight the importance of investigating the mechanism of injury when someone presents to ED, as injuries may be related to physical abuse or domestic violence, especially in women. Second, while ED presentations in adulthood cannot undo the experiences of CM, contact with the healthcare system may offer an opportunity to minimise the enduring burden of disease CM propagates (Gateway, 2023). Indeed, tertiary preventive support for individuals affected by CM should aim to reduce and prevent further harms; prevent intergenerational transmission of CM; and provide holistic, ongoing support that is trauma-informed in recognition of their experiences of CM and its enduring consequences (Widom et al., 2015). Third, our findings present an avenue for future research to better understand why sex differences occur in relation to ED injury presentations, which may then inform the development and implementation of targeted interventions. Finally, though the majority of ED injury presentations are minor (Dinh et al., 2017), they still represent significant economic burden to healthcare services. With the prospective data involved in this study, this study provides evidence of the broader economic cost of CM, illustrating that we need to address the root cause of the issue, not just its symptoms.

The results of this study also need to be considered within its limitations. Firstly, the criteria for reporting and substantiating CM in the 1980s and 90s may not reflect current reporting methods. Furthermore, because self-reported and agency-reported reported CM do not always represent the same population of people (Kisely et al., 2021), the generalisability of these results to self-reported CM may be limited. Secondly, the outcomes in this study were based on administrative data, which might be influenced by recording bias. More specifically, we cannot exclude the possibility that presentations for DSH or suicide may have been classified as non-deliberate, and reliance on agency reported CM is likely to result in underestimated cases of CM (Gilbert et al., 2009). Thirdly, limitations of the methods of reporting in the ED mean that we could not determine the exact causes of injuries. Lastly, because of the a priori nature of our hypothesis, correction for multiple comparisons was less appropriate (Armstrong, 2014; Barnett et al., 2022; Perneger, 1998), and therefore not conducted. However, the likelihood of false positive results is unlikely given the strong effect sizes seen.

In conclusion, although associations between presentations at the ED for injuries were associated with several forms of notified and substantiated CM, upon stratification these associations were more pronounced in women, with women having significantly higher odds of presenting to the ED for injuries if they had notified or substantiated physical abuse, substantiated emotional abuse, or more than two types of substantiated abuse. The mechanisms for these associations are likely multifaceted and complex, warranting further exploration. Future investigations should aim to stratify results by sex to corroborate these findings. Public health professionals, as well as hospital practitioners should take note of these findings to inform targeted CM prevention interventions and improved care.

## Supplemental Material

Supplemental Material - Emergency Department Presentations for Injuries Following Agency-Notified Child Maltreatment: Results From the Childhood Adversity and Lifetime Morbidity (CALM) Study Supplemental Material for Emergency Department Presentations for Injuries Following Agency-Notified Child Maltreatment: Results From the Childhood Adversity and Lifetime Morbidity (CALM) Study by Mike Trott, Claudia Bull, Urska Arnautovska, Dan Siskind, Nicola Warren, Jake M. Najman, and Steve Kisely in Child Maltreatment.

## Data Availability

Due to privacy, ethical and legal considerations, the administrative health data cannot be shared without direct approval from relevant data custodians and the Office of Research and Innovation of Queensland Health. Contact details for Queensland Health custodians can be found at https://www.health.qld.gov.au/__data/assets/pdf_file/0034/843199/data_custodian_list.pdf
