# Supplementary material for: Emergency Department Presentations for Injuries Following Agency-Notified Child Maltreatment: Results From the Childhood Adversity and Lifetime Morbidity (CALM) Study
Source: Child Maltreat. 2024 Jun 20;30(4):603–11. doi: 10.1177/10775595241264009 (PMC12495111; doi:10.1177/10775595241264009)
Supplement: Supplemental Material - Emergency Department Presentations for Injuries Following Agency-Notified Child Maltreatment: Results From the Childhood Adversity and Lifetime Morbidity (CALM) Study [file sj-pdf-1-cmx-10.1177_10775595241264009.pdf]

Supplementary Table 1: Odds of ED presentations between 25-39 years of age for injury following previous CM with sensitivity analyses

	**Overall†**		**Male††**		**Female††**	
	Core analysis	PS analysis	Core analysis	PS analysis	Core analysis	PS analysis
Any agency-reported notifications of CM	1.57 (1.32-1.87)	1.56 (1.32-1.86)	1.40 (1.09-1.78)	1.40 (1.10-1.76)	1.74 (1.37-2.22)	1.76 (1.38-2.24)
Physical Abuse	1.56 (1.25-1.94)	1.53 (1.23-1.90)	1.23 (0.91-1.66)	1.24 (0.92-1.67)	1.99 (1.45-2.73)	2.00 (1.46-2.75)
Emotional Abuse	1.54 (1.23-1.93)	1.54 (1.23-1.93)	1.34 (0.98-1.83)	1.34 (0.98-1.84)	1.77 (1.29-2.45)	1.80 (1.30-2.48)
Neglect	1.62 (1.30-2.01)	1.63 (1.31-2.02)	1.54 (1.14-2.08)	1.55 (1.14-2.09)	1.69 (1.24-2.31)	1.72 (1.26-2.35)
Sexual abuse	1.43 (1.07-1.92)	1.43 (1.07-1.91)	1.29 (0.75-2.22)	1.29 (0.75-2.22)	1.48 (1.04-2.09)	1.49 (1.05-2.11)
Two or more sub-types	1.58 (1.28-1.95)	1.58 (1.28-1.94)	1.37 (1.02-1.83)	1.37 (1.03-1.84)	1.82 (1.35-2.45)	1.84 (1.37-2.48)
Any substantiated notifications of CM	1.41 (1.14-1.75)	1.42 (1.15-1.75)	1.23 (0.92-1.66)	1.24 (0.92-1.66)	1.60 (1.19-2.16)	1.62 (1.20-2.19)
Physical Abuse	1.47 (1.10-1.95)	1.47 (1.10-1.94)	1.13 (0.76-1.66)	1.13 (0.77-1.67)	1.96 (1.30-2.96)	1.98 (1.31-3.00)

Emotional Abuse	1.52 (1.14-2.03)	1.52 (1.14-2.02)	1.16 (0.77-1.74)	1.16 (0.77-1.75)	1.98 (1.32-2.95)	2.01 (1.24-3.00)
Neglect	1.60 (1.19-2.15)	1.61 (1.21-2.16)	1.32 (0.88-1.99)	1.33 (0.89-1.99)	1.91 (1.25-2.90)	1.95 (1.28-2.96)
Sexual abuse	1.15 (0.77-1.71)	1.15 (0.78-1.71)	0.89 (0.41-1.92)	0.89 (0.41-1.92)	1.23 (0.78-1.96)	1.24 (0.78-1.97)
Two or more sub-types	1.41 (1.06-1.87)	1.40 (1.06-1.85)	1.06 (0.71-1.60)	1.07 (0.71-1.60)	1.79 (1.22-2.63)	1.82 (1.24-2.67)

All results are shown as adjusted odds ratios (95% CI); ED = Emergency Department; CM = Childhood Maltreatment; PS=propensity score; † adjusted for parental income; gender; parental race; parental relationship status; †† adjusted for parental income; parental race; parental relationship status
